# Accurate total consumer price index forecasting with data augmentation, multivariate features, and sentiment analysis: A case study in Korea

**DOI:** 10.1371/journal.pone.0321530

**Published:** 2025-05-13

**Authors:** Injae Seo, Minkyoung Kim, Jong Wook Kim, Beakcheol Jang

**Affiliations:** 1 Graduate School of Information, Yonsei University, Seoul, Republic of South Korea; 2 Department of Computer Science, Sangmyung University, Seoul, Republic of South Korea; The University of Tokyo: Tokyo Daigaku, JAPAN

## Abstract

The Consumer Price Index (CPI) is a key economic indicator used by policymakers worldwide to monitor inflation and guide monetary policy decisions. In Korea, the CPI significantly impacts decisions on interest rates, fiscal policy frameworks, and the Bank of Korea’s strategies for economic stability. Given its importance, accurately forecasting the Total CPI is crucial for informed decision-making. Achieving accurate estimation, however, presents several challenges. First, the Korean Total CPI is calculated as a weighted sum of 462 items grouped into 12 categories of goods and services. This heterogeneity makes it difficult to account for all variations in consumer behavior and price dynamics. Second, the monthly frequency of CPI data results in a relatively sparse time series, limiting the performance of the analysis. Furthermore, external factors such as policy changes and pandemics add further volatility to the CPI. To address these challenges, we propose a novel framework consisting of four key components: (1) a hybrid Convolutional Neural Network-Long Short-Term Memory mechanism designed to capture complex patterns in CPI data, enhancing estimation accuracy; (2) multivariate inputs that incorporate CPI component indices alongside auxiliary variables for richer contextual information; (3) data augmentation through linear interpolation to convert monthly data into daily data, optimizing it for highly parametrized deep learning models; and (4) sentiment index derived from Korean CPI-related news articles, providing insights into external factors influencing CPI fluctuations. Experimental results demonstrate that the proposed model outperforms existing approaches in CPI prediction, as evidenced by lower RMSE values. This improved accuracy has the potential to support the development of more timely and effective economic policies.

## Glossary of Terms

**Table:** 

Acronym/Term	Definition
BERT	Bidirectional Encoder Representations from Transformers: A deep learning model designed for natural language processing tasks, enabling contextual understanding of text.
KLUE-BERT	Korean Language Understanding Evaluation BERT: A BERT model fine-tuned for Korean language tasks as part of the KLUE benchmark.
KoBERT	Korean Bidirectional Encoder Representations from Transformers: A BERT-based model pre-trained for Korean-specific language understanding tasks.
CPI	Consumer Price Index: A measure representing the average price level of a basket of consumer goods and services. In Sects 1 and 2, CPI is discussed in general terms. From Sect 3 onward, unless otherwise stated, this paper refers specifically to the Korean Total CPI (2015 = 100).
CPI indices	**Total CPI:** The aggregate Consumer Price Index, is calculated as the weighted sum of its component indices.
	**Component Indices:** Specific indices representing individual categories or subsets within the overall CPI, such as sector-specific price changes.
	**Auxiliary Indices:** Supplementary indices that provide additional context or insights into the behavior and trends of the primary CPI.
Sentiment Index	daily sentiment scores extracted from Korean news articles related to CPI unless otherwise noted.
FRED	Federal Reserve Economic Data: A publicly accessible database providing economic and financial data for research and analysis.
ANN	Artificial Neural Network: A machine learning model inspired by biological neural networks, used for various predictive tasks.
CNN	Convolutional Neural Network. Can refer to 2D or 1D CNN while 1D CNN was used in our framework.
ARIMA	Autoregressive Integrated Moving Average: A classical statistical model for time series forecasting that models dependencies between observations.
1D CNN	1-Dimensional Convolutional Neural Network: A neural network architecture designed to process one-dimensional data, such as time series.
CNN-LSTM	Convolutional Neural Network-Long Short-Term Memory: A hybrid deep learning model that integrates 1D CNN for feature extraction with LSTM for capturing sequential dependencies, thereby improving the accuracy of multivariate time series forecasting.
AttentionLSTM	Attention Long Short-Term Memory Network: An enhanced LSTM model with an attention mechanism to prioritize relevant time steps in sequential data, improving prediction accuracy.
GRU	Gated Recurrent Unit: A recurrent neural network variant that efficiently handles sequential data by mitigating the vanishing gradient problem.
LSTM	Long Short-Term Memory Network: A type of recurrent neural network architecture designed to process and learn dependencies in sequential data.
SVR/SVM	Support Vector Regression/Support Vector Machine: SVR is a regression algorithm based on SVM principles, designed to predict continuous outcomes by fitting a hyperplane within a specified margin in high-dimensional space. SVM is primarily a classification algorithm that identifies the optimal hyperplane to separate classes.
FFNN	Feedforward Neural Network with 2 Fully Connected (FC) Layers: A straightforward neural network architecture for time-series forecasting. It uses two sequential fully connected layers to capture complex nonlinear patterns in the data.
XGBoost	Extreme Gradient Boosting: A scalable tree-based algorithm used for regression and classification tasks, including time series forecasting.
NLP	Natural Language Processing: A field of AI focused on understanding, interpreting, and generating human language.
MAE	Mean Absolute Error: A metric less sensitive to outliers that calculates the average magnitude of residuals.
MAPE	Mean Absolute Percentage Error: A metric quantifying prediction errors as a percentage of actual values.
RMSE	Root Mean Squared Error: A model evaluation metric measuring the square root of the average squared residuals.
NRMSE	RMSE normalized by the standard deviation of the actual data.
SMAPE	Symmetric Mean Absolute Percentage Error: A metric addressing overestimation in percentage errors by symmetrically evaluating errors relative to actual values.
TP, FP, TN, FN	**True Positives (TP):** Correctly identified positive cases.
	**False Positives (FP):** Cases incorrectly classified as positive.
	**True Negatives (TN):** Correctly identified negative cases.
	**False Negatives (FN):** Cases incorrectly classified as negative.
F1 Score	A metric for evaluating model performance, representing the harmonic mean of precision and recall.

## 1. Introduction

The COVID-19 pandemic has profoundly impacted global economies, leading to rapid inflation characterized by continuous price increases. Inflation erodes purchasing power, reduces currency value, and destabilizes daily life, ultimately undermining economic stability [1]. The Consumer Price Index (CPI) serves as a critical economic indicator, reflecting inflationary trends and providing insights for policymakers and stakeholders. Accurate CPI forecasting is vital for effective economic planning and decision-making. For example, in South Korea, the National Pension Service adjusts pension payments based on CPI fluctuations to ensure fair compensation, while the Bank of Korea employs CPI for inflation targeting to stabilize the economy [2].

Considering the fundamental importance of the CPI, reliable forecasting is crucial. Nevertheless, forecasting CPI is fraught with challenges. For instance, external events such as the pandemic can significantly disrupt CPI dynamics. During the pandemic, consumption patterns shifted drastically, with heightened demand for essential goods (e.g., groceries, protective equipment) and reduced demand for travel-related products [3]. Supply chain disruptions and economic recovery intensified inflationary pressures, particularly in sectors like transportation, food, and accommodation [4, 5]. The adoption of expansionary fiscal and monetary policies further contributed to inflationary pressures [6].

The complexity of forecasting CPI also arises from its composition of multiple sub-indices, each governed by distinct dynamics. In South Korea, CPI is calculated monthly based on 462 representative items across 12 categories, such as food, housing, and transportation. Sector-specific fluctuations can often be masked in the Total CPI; for instance, rising housing costs may be offset by falling food prices, resulting in a stable overall CPI. Utilizing CPI components is thus essential for accurate forecasting.

Furthermore, the limited frequency of CPI data–typically measured monthly–yields only 12 observations per year, making time-series forecasting more challenging. External factors, such as pandemics, policy shifts, and natural disasters, further complicate these efforts. Incorporating supplementary data sources, such as sentiment analysis of news articles, can offer valuable context for understanding and predicting CPI fluctuations.

To address these challenges, we propose a hybrid Convolutional Neural Network-Long Short-Term Memory (CNN-LSTM) model that leverages multivariate data and sentiment analysis. The model introduces the following key components:

- **Multivariate input:** By integrating component and auxiliary CPI indices and sentiment index derived from news articles, the model captures sector-specific dynamics and external influences on Korean Total CPI [7].
- **Data augmentation:** Linear interpolation generates daily data points from monthly observations, addressing data scarcity, crucial for deep learning-based models.
- **Sentiment analysis:** Sentiment scores derived from news articles related to global events provide additional context, enhancing the model’s responsiveness to external shocks
- **CNN-LSTM hybrid architecture:** The CNN can extract features from multivariate data, while the LSTM captures sequential dependencies, enhancing prediction accuracy [8].

Extensive experiments demonstrate that our model outperforms existing methods, achieving superior predictive performance by combining multivariate inputs, data augmentation, sentiment analysis, and a CNN-LSTM mechanism.

Sect 2 reviews the related work on CPI prediction. Sect 3 describes the methodology of our model, followed by the experimental setup in Sect 4. Sect 5 presents the results, and Sect 6 provides the conclusion.

## 2. Related work

### 2.1. Traditional and advanced approaches for CPI Forecasting

Forecasting the CPI has long relied on traditional statistical methods, with Autoregressive Integrated Moving Average (ARIMA) being one of the most widely used. Zhang *et al*. [9] demonstrated ARIMA’s effectiveness in predicting macroeconomic indicators, while Ahmar *et al*.[10] applied it to model CPI in Indonesia, showcasing its accuracy in practical economic scenarios. Similarly, Shinkarenko *et al*. [11] analyzed CPI dynamics in Ukraine with ARIMA, illustrating its ability to track consumer price trends. Hybrid approaches have also been explored; for example, Liu [12] combined ARIMA with LSTM models to predict China’s CPI, finding that this combination improved forecasting accuracy by leveraging the strengths of both methods.

Machine learning methods such as Markov Chain [13], Support Vector Regression (SVR) [14]/SVM [15] have also been used for CPI forecasting. However, these approaches typically rely on univariate inputs, which limits their ability to model more complex relationships.

The development of deep learning methods has opened up new possibilities for CPI forecasting. Early studies, such as those by Moshiri *et al*. [16], Chen *et al*. [17], and Szafranek *et al*. [18], showed that neural networks often outperformed traditional statistical methods. Cross-country analyses by Mcadam *et al*. [19] and Choudhary *et al*. [20] further emphasized the advantages of these models for inflation forecasting. Nakamura *et al*. [21] found that Feedforward Neural Networks (FFNNs) were particularly effective in predicting U.S. CPI on a quarterly basis, outperforming autoregressive baselines. Building on this, Almosova *et al*. [22] Zahara *et al*. [7] applied LSTM networks for inflation forecasting, achieving better accuracy than traditional models. Zheng *et al*. [23] pushed this further by combining penalized regression with mixed-frequency data sampling and incorporating media and internet data, which led to significant improvements in forecasting performance. Recent studies have explored more complex deep-learning approaches. Similarly, Barkan *et al*. developed a hierarchical recurrent neural network to forecast disaggregated CPI components, achieving better results than baseline models [24].

Overall, these studies reflect the growing importance of advanced computational techniques in CPI forecasting. Nevertheless, most methods have primarily focused on univariate data. No studies have directly addressed the sparse frequency data of CPI, and most studies relied on simplistic models.

### 2.2. Hybrid forecasting model utilizing CNN or LSTM models

Recurrent Neural Network (RNN)-based architectures effectively train sequential data by processing inputs and outputs in sequence, leveraging historical information from hidden layers. However, as the input time-series length increases, traditional RNNs struggle with long-term dependencies, leading to the loss of historical information. To address this, Hochreiter *et al*. [25] proposed the LSTM model, which has shown promising results in time series prediction [26, 27].

For instance, Li *et al*. [28] demonstrated that LSTM outperformed SVM and linear regression models in predicting the Chinese stock market, even when incorporating sentiment analysis. However, LSTM’s ability to fully capture complex variable interactions is limited, as its strength lies in maintaining temporal dependencies.

Recent studies have shown that CNN-based models can address these limitations in time series prediction. For example, in the financial domain, Hoseinzade *et al*. [29] proposed CNNpred, a framework for stock market prediction that uses feature extraction and cross-market correlations, achieving significant improvements over traditional methods.

To leverage the strengths of both approaches, hybrid CNN-LSTM models have been proposed. CNNs are employed for feature extraction, while LSTMs handle the temporal dependencies inherent in time series data. Livieris *et al*. [30] and others [31, 32] demonstrated the effectiveness of this hybrid approach in gold price forecasting, where CNN extracts key features, and LSTM predicts future values. These studies underscore the advantages of combining 1-dimensional (1D) CNN and LSTM for capturing complex interactions and sequential patterns, resulting in improved prediction accuracy.

Nevertheless, only a few of these hybrid models have been applied to CPI forecasting. Moreover, they failed to acknowledge the significance of handling monthly frequency data effectively.

### 2.3. Sentiment analysis for financial forecasting

Sentiment analysis has become a pivotal tool in financial forecasting, leveraging textual data to enhance prediction accuracy. For example, Nia *et al*. [33] used Twitter sentiment analysis and machine learning to examine macroeconomic responses to COVID-19, revealing disparities in unemployment and inflation control across income-level groups globally. Similarly, Song *et al*. [34] demonstrated that incorporating social media sentiment into RNN models improved the prediction accuracy of infectious disease cases, highlighting the qualitative impact of public opinion on disease spread.

Shapiro *et al*. [35] measured economic news sentiment by combining lexicon-based scoring and machine learning, explicitly accounting for linguistic nuances such as negation, and constructing a sentiment time series index with predictive power for macroeconomic variables and consumer sentiment. In contrast, Li *et al*. [36] combined Naive Bayes sentiment scoring with LSTM networks and Support Vector Machines (SVMs) to predict Chinese stock market prices, showing that integrating sentiment analysis with external events can significantly enhance forecasting accuracy.

Building on these approaches, De Oliveira Carrosia *et al*. [8] introduced sentiment-driven investment strategies for the Brazilian stock market, using CNN-based analysis of financial news in Portuguese. Their study demonstrated superior profitability over traditional strategies like Random Walk and Buy and Hold in both short- and long-term scenarios. Similarly, Li *et al*. [37] incorporated investor sentiment into deep learning models, further validating the potential of sentiment analysis to improve stock price predictions in the Chinese market.

Meanwhile, recent developments in Korean Natural Language Processing (NLP) have introduced pre-trained language models tailored to the Korean language, such as Korean Bidirectional Encoder Representations from Transformers (KoBERT) [38] and KLUE-BERT [39]. KoBERT is trained on a large corpus of Korean text, including Wikipedia and news articles. It utilizes a SentencePiece tokenizer and consistently outperforms multilingual BERT on Korean NLP tasks [40].

KLUE-BERT [39] is another model designed for diverse NLP tasks, being trained on a 62GB corpus comprising of diverse format: formal articles, web content, and colloquial text. With its morpheme-based tokenization and 111M parameters, KLUE-BERT has demonstrated superior performance on KLUE benchmark tasks.

Despite these advances, the use of sentiment analysis in Korean financial forecasting remains underexplored. While research has focused on general sentiment-based forecasting, specific applications such as predicting the CPI in Korea are lacking. Korean NLP pre-trained models like KoBERT and KLUE-BERT have significantly improved sentiment analysis capabilities, yet their potential for CPI forecasting and other time-series tasks has not been fully realized. Furthermore, existing studies rarely incorporate data augmentation techniques, or use more advanced deep learning-based models like CNN-LSTM.

Further, the limited application of data augmentation techniques in sentiment-based forecasting further underscores this gap. Current research largely relies on traditional forecasting methods, overlooking the potential of augmentation to improve model performance, particularly in multivariate scenarios. Exploring these techniques could lead to significant advancements in prediction accuracy for tasks such as CPI forecasting and other time-series forecasting challenges in the Korean financial domain. By addressing these limitations, future research can unlock new opportunities for integrating sentiment analysis into advanced forecasting frameworks.

## 3. Methodology

This study integrates numerical features (CPI indices) and textual data (CPI-related news articles) through a structured preprocessing pipeline. First, variable selection is applied to both numerical and textual data (Sect 3.1), followed by text processing techniques such as summarization and sentiment index calculation (Sect 3.2). The processed data is then augmented to enhance model robustness (Sect 3.3) before constructing a sliding window dataset for sequential learning (Sect 3.4). Finally, the prepared dataset is used for CNN-LSTM model training and evaluation (Sect 3.5).

**Fig 1:**
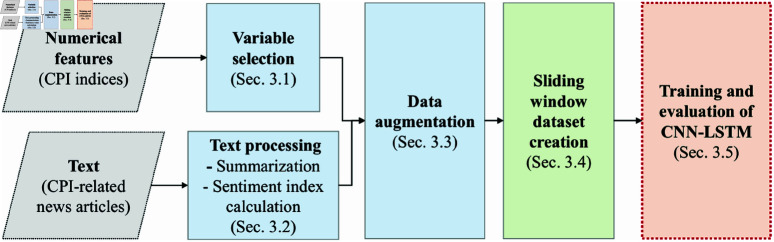
The data preprocessing and modeling pipeline for CPI forecasting.

Fig 1 provides an overview of this pipeline, illustrating the key steps from feature selection to model training. The following subsections elaborate on each stage in detail.

### 3.1. Collection and selection of CPI-related numerical features

The CPI-related dataset was obtained using the Federal Reserve Economic Data (FRED) API (https://github.com/mortada/fredapi) by searching for ‘South Korea CPI 2015:100.’ FRED, managed by the Federal Reserve Bank of St. Louis, offers an extensive repository of economic data, including international statistics.

The CPI-related indices are expressed as a relative index, with the base year set to 2015 (2015 = 100). The methodology ensures the indices are relevant to consumer behavior. The Korean Total CPI is calculated using a modified Laspeyres formula [41], a widely used method for computing price indices. This method calculates the cost of purchasing a fixed basket of goods and services in the current period relative to the base period, reflecting changes in price levels over time. The formula considers the prices of individual items from both the base and current periods, weighted by their respective consumption shares in the base period, ensuring that the calculated index accurately represents consumer spending patterns and inflation trends. The Laspeyres formula can be expressed as:

$$\text{Total CPI}=\frac{\sum_{i=1}^{n}p_{i,t}q_{i,0}}{\sum_{i=1}^{n}p_{i,0}q_{i,0}}\times 100$$ (1)

In this formula, *p*_*i*,*t*_ represents the price of item *i* in the current period, *p*_*i*,0_ represents the price of item *i* in the base period, and *q*_*i*,0_ denotes the quantity of item *i* consumed in the base period. The denominator reflects the total cost of the basket in the base period, while the numerator captures the cost in the current period [42].

The Korean Total CPI (FRED ID ‘KORCPIALLMINMEI’) serves as a comprehensive measure of consumer prices, encompassing 462 representative items that account for 100% of the overall weight. It is derived as a weighted sum of its component indices, which include categories like housing, transportation, and food, as detailed in Table 1. These components are categorized based on their contribution to consumer expenditures.

**Table 1:** CPI-related feature dataset for South Korea.

FRED ID	Title	Amt. (%)	Coef.	Selected
Primary index				
KORCPIALLMINMEI	Total CPI	462 (100.0%)	-	
Component indices				
KORCP010000IXOBM	Food and non-alcoholic beverages	133 (28.8%)	4.79	
KORCP020000IXOBM	Alcoholic beverages, tobacco and narcotics	7 (1.5%)	0.28	
KORCP020000IXOBM	Clothing and footwear	30 (6.5%)	0.32	
KORCP040000IXOBM	Housing, water, electricity, and fuel	16 (3.5%)	3.29	
KORCP050000IXOBM	Household goods and services	49 (10.6%)	-	
KORCP060000IXOBM	Health	33 (7.1%)	-	
KORCP070000IXOBM	Transportation	32 (6.9%)	1.51	
KORCP080000IXOBM	Communication	6 (1.3%)	-	
KORCP090000IXOBM	Recreation and culture	56 (12.1%)	-	
KORCP100000IXOBM	Education	20 (4.3%)	0.03	
KORCP110000IXOBM	Restaurants and hotels	44 (9.5%)	-	
KORCP120000IXOBM	Miscellaneous goods and services	36 (7.8%)	-	
Auxiliary indices				
KORCPICORMINMEI	All items (non-food non-energy)	-	6.61	
KORCPIENGMINMEI	Energy	-	-	
KORCPGRSE01IXOBM	Services	-	0.36	
KORCPGRLH02IXOBM	Services less housing	-	2.47	
KORCPGRHO02IXOBM	Housing excluding imputed rentals for housing	-	-	
KORCP040100IXOBM	Actual rentals for housing	-	0.14	
KORCP040400IXOBM	Water supply and misc. services relating to dwelling	-	-	
KORCP040500IXOBM	Electricity, gas and other fuels	-	-	
KORCP040300IXOBM	Maintenance and repair of the dwelling	-	-	

The component indices represent major expenditure categories, such as *Food and non-alcoholic beverages* (FRED ID ‘KORCP010000IXOBM,’ 28.8%), *Housing, water, electricity, and fuel* (‘KORCP040000IXOBM,’ 3.5%), along with *Health*, *Transportation*, and *Education*. Each component is weighted according to its relative contribution to total consumer spending. Auxiliary indices, such as *All items (non-food non-energy)* (‘KORCPICORMINMEI’) and *Services less housing* (‘KORCPGRLH02IXOBM’), provide additional granularity for specific analytical needs.

We included 22 indices for analysis: Total CPI, 12 key component indices, and 9 auxiliary indices. Monthly data spanning January 2010 to November 2021 was utilized. Feature importance was assessed using Lasso regression [43], which identifies significant variables by penalizing less influential ones. A 30% threshold was applied, and the non-zero coefficients are summarized in Table 1. Using Lasso regression, 2 component indices and 2 auxiliary indices were identified as significant contributors to CPI variations. Notable components include *Food and non-alcoholic beverages*, as well as *Housing, water, electricity, and fuel*.

**Fig 2:**
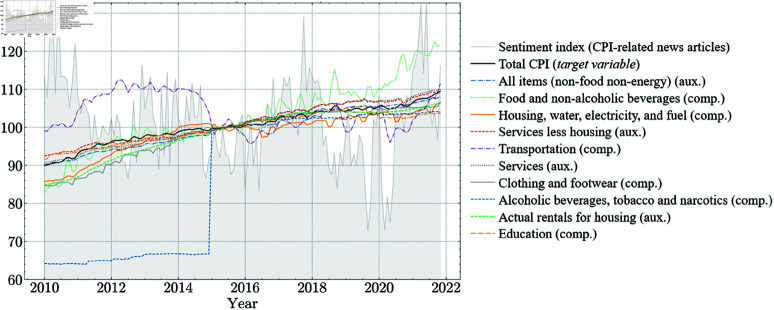
Actual values of variables with non-zero Lasso coefficients and the sentiment index. The legend orders variables by descending the Lasso coefficient, with the most influential following Total CPI.

Fig 2 presents the actual values of non-zero coefficient variables alongside the sentiment index. The legend orders variables by descending the Lasso coefficient, with the most influential variable following Total CPI. Auxiliary and component variables are intermixed, indicating that a higher component weight does not necessarily correspond to a higher coefficient. Notably, *Transportation* and *Alcoholic Beverages* exhibit distinct trends yet remain influential. The shaded area represents the sentiment index. Corresponding Lasso coefficient values are provided in Table 1.

Notably, component indices such as *Food and housing* exhibit greater variability compared to categories like *Education* and *Communication*, reflecting their stronger influence on the Total CPI. The sentiment score also aligns with significant CPI shifts, particularly during external shocks like the COVID-19 pandemic. These patterns underscore the value of incorporating both component indices and sentiment scores to improve CPI forecasting accuracy.

### 3.2. News articles collection and sentiment extraction

News articles related to the CPI were collected from Naver News (https://www.news.naver.com) using ‘CPI’ as the search keyword. These articles were used to represent external events in the sentiment analysis. The corpus comprised articles published between January 1, 2010, and November 21, 2021. Each article is accompanied by the date of publication, title, text, and source. Naver News is a platform that aggregates articles from various Korean newspaper publishers, offering a centralized repository of diverse news content. The total number of articles collected was 213,983, spanning 4,323 days. The data were then processed into a single variable for CPI forecasting through sentiment analysis and index calculation.

#### 3.2.1. Summary using TextRank.

Before calculating the sentiment index, we created a summary for each article using TextRank [44] as analyzing the entire article often incorporates irrelevant content into the sentiment score, complicating the analysis of the core message. As suggested by Mihalcea *et al*. [44], we extracted three key sentences per article. TextRank is an unsupervised, graph-based ranking model for text processing that can be effectively used for extractive summarization [45]. It works by constructing a graph representation of sentences in the text, where each sentence is a node, and edges between sentences are weighted based on their similarity [46]. The algorithm then applies a graph-based ranking algorithm similar to PageRank [47] to identify the most important sentences [44]. The TextRank algorithm for sentence extraction follows these steps [46]: (1) Split the text into sentences; (2) Create a graph where sentences are nodes; (3) Calculate the similarity between sentences to create weighted edges; (4) Apply the PageRank [47] algorithm to rank sentences; (5) Select the top-ranked sentences as the summary.

By extracting only the most representative sentences, TextRank helps focus on the core content of the article, potentially improving the accuracy of sentiment analysis by reducing noise from less relevant sentences [45].

#### 3.2.2. Sentiment index calculation.

Prior to computing the sentiment index from summaries, we first conducted a preliminary study to determine the most suitable sentiment classifier. This study was performed on the Korean-translated version of the Finance Phrasebank dataset [48] using two embedding-based classifier models: KoBERT [40] and KLUE-BERT [39].

The Finance Phrasebank dataset [48] consists of 4,846 English sentences manually labeled as positive, neutral, or negative by financial experts. The label proportions in the translated dataset were 28.22% positive, 59.27% neutral, and 12.51% negative.

KoBERT is a pre-trained BERT model specifically designed for the Korean language. It is trained on Korean datasets, which enhances its performance on tasks involving Korean text compared to multilingual models. KLUE-BERT [39], on the other hand, is part of the KLUE benchmark designed for advanced NLP tasks in Korean. Trained on a large corpus of Korean text, KLUE-BERT effectively captures the linguistic nuances of the Korean language. Sentiment classifications–positive, neutral, or negative–were evaluated based on the F1 score.

Classifier performance was assessed using the F1 score, which balances Precision and Recall. These metrics are defined as follows:

$$\text{Precision}=\frac{\text{TP}}{\text{TP}+\text{FP}},\;\text{Recall}=\frac{\text{TP}}{\text{TP}+\text{FN}},$$ (2)

$$\text{F1 Score}=2\times \frac{\text{Precision}\times \text{Recall}}{\text{Precision}+\text{Recall}},$$ (3)

where TP, FP, and FN represent true positives, false positives, and false negatives, respectively. The F1 score is particularly useful for imbalanced datasets, as it equally emphasizes Precision and Recall.

In preliminary experiments, KLUE-BERT demonstrated superior performance over KoBERT [49]. Table 2 shows the evaluation results, where KLUE-BERT achieved an F1 score of 0.852 compared to KoBERT’s 0.434. This led us to adopt KLUE-BERT as the sentiment classifier for calculating the sentiment index, which serves as an input to our main forecasting model.

**Table 2:** Evaluation results for sentiment classifier models.

Pre-trained Model	Accuracy	Precision	Recall	F1 Score
KLUE-BERT	0.852	0.852	0.852	0.852
KoBERT	0.436	0.505	0.436	0.434

Using KLUE-BERT, we performed sentiment analysis on 208,455 CPI-related news articles spanning January 1, 2010, to November 1, 2021. The analysis categorized the articles into 70,324 positive, 70,348 neutral, and 67,783 negative instances, providing a sentiment profile of the dataset.

Then, we constructed a daily sentiment index from the classes of each news article in the dataset as positive, neutral, or negative. The sentiment score for each day was calculated using a formula originally proposed by Antweiler *et al*. [50], which has been widely applied in sentiment analysis studies [14, 51, 52]:

$$\text{Sentiment score}=\frac{N_{t}^{\text{pos}}-N_{t}^{\text{neg}}}{N_{t}^{\text{pos}}+N_{t}^{\text{neg}}},$$ (4)

where $N_{t}^{\text{pos}}$ and $N_{t}^{\text{neg}}$ are the numbers of positive and negative news items, respectively, on the day *t*. The sentiment score, bound between -1 and +1, indicates daily sentiment trends.

#### 3.2.3. Assessment of sentiment index as a predictor of CPI.

This analysis evaluates the effectiveness of our sentiment index as a predictor of CPI movements by addressing two key questions: whether our methodology for calculating the sentiment index is theoretically sound, and whether the sentiment index can serve as a reliable leading indicator for CPI movements.

To address these questions, we conducted cross-correlation analyses of three relationships. First, we examined the relationship between COVID-19 cases and the sentiment index to validate our measurement methodology. We then evaluated the sentiment index against the lagged Total CPI to assess its predictive capabilities and investigated the feedback effects by analyzing the Total CPI relative to the lagged sentiment index.

The cross-correlation coefficient between two time series *x*_*t*_ and *y*_*t*_ is defined as:

$$\rho_{xy}(\tau )=\rho (x_{t-\tau },y_{t})=\frac{\text{Cov}(x_{t-\tau },y_{t})}{\sqrt{\text{Var}(x_{t-\tau })\cdot \text{Var}(y_{t})}}=\frac{\gamma_{xy}(\tau )}{\sigma_{x}\sigma_{y}},$$ (5)

where $x_{t-\tau }$ represents the lagged value of the time series *x* by τ time steps, and *y*_*t*_ represents the current value. We applied a 5-month moving average smoothing to the original noisy data, which helped capture long-term patterns more effectively.

**Fig 3:**
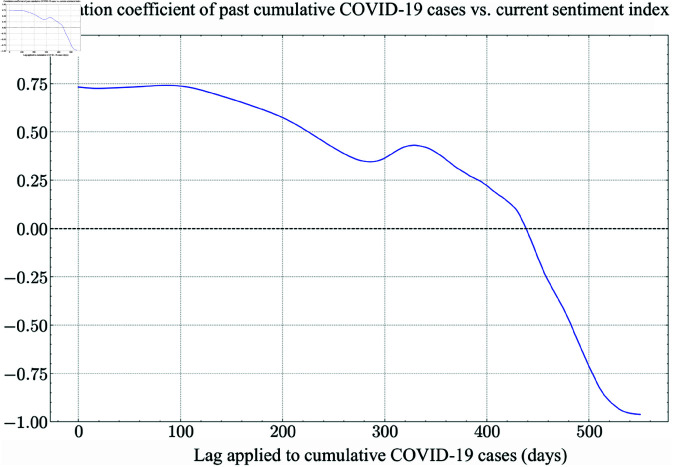
Correlation between past cumulative COVID-19 cases and the current sentiment index. The long-term negative trend suggests a delayed adverse effect on sentiment.

**Fig 4:**
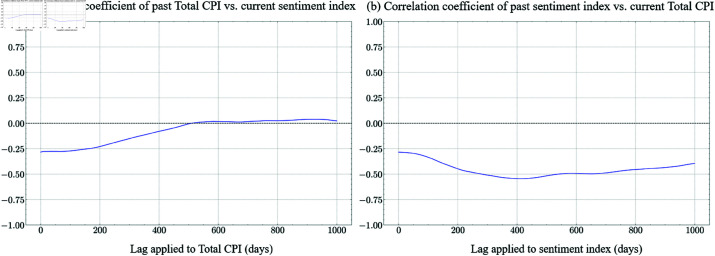
Correlation between Total CPI and the sentiment index over time. (a) The correlation between past Total CPI and the current sentiment index is weak overall. (b) The correlation between past sentiment and present Total CPI remains negative.

In Fig 3 and Fig 4(a) and Fig 4(b), the x-axis represents the time lag, while the y-axis indicates the corresponding correlation coefficient values. Fig 3 illustrates the correlation between past cumulative COVID-19 cases and the sentiment index as a function of the applied time lag, measured in days. Fig 4(a) and Fig 4(b) each represent the correlation between past Total CPI and the current sentiment index, and the correlation between the past sentiment index and the current Total CPI, respectively, as a function of lag in days.

Fig 3 shows that the correlation between the sentiment index and lagged cumulative cases varies significantly depending on the lag period, with a maximum lag of 550 days. At zero lag, the correlation is strongly positive with a coefficient of +0.80, suggesting that higher recent COVID-19 case counts coincide with higher sentiment. This positive relationship persists but weakens over time. Around 480 days, the correlation crosses zero, and as the lag extends beyond 500 days, the relationship becomes negative, reaching approximately -0.75 at 500 days and plunging to less than -0.80 at 550 days. While it might seem counterintuitive for sentiment to be positively correlated with case counts in the short term, this pattern could be influenced by factors such as heightened public awareness, media narratives, or government actions that shaped sentiment during outbreaks. However, as time progresses, older case counts appear to have an inverse relationship with present sentiment, suggesting a delayed negative association. It is noteworthy that although cumulative COVID-19 cases strongly correlate with the Total CPI (+0.94 at lag 0), this relationship was excluded as a primary predictor due to the pandemic’s temporary nature and our target dataset length.

In Fig 4(a), the correlation coefficients between the sentiment index and the lagged Total CPI over 0 to 1000 days are too weak to be deemed significant. At a lag of 0 days, the correlation is around -0.3, indicating that when the Total CPI is high on a given day, sentiment tends to be lower on the same day. As the lag increases to 200 days, the correlation improves to about -0.15, showing a weaker negative relationship. By 400 days, the correlation approaches 0.0, suggesting that the influence of past Total CPI values on current sentiment diminishes over time. Between 600 and 800 days, the correlation fluctuates slightly between -0.05 and 0.0, and by 1000 days, it stabilizes near 0.0. This pattern suggests that while more recent Total CPI values exhibit a negative association with sentiment, this effect weakens as the lag increases. Beyond approximately 400 days, past values of the Total CPI have little to no measurable relationship with present sentiment.

In Fig 4(b), the correlation remains consistently negative across all time lags. At a lag of zero, the correlation coefficient is approximately -0.28. As the lag increases, the correlation becomes more negative, reaching its lowest point near -0.5 at around 400 days. Beyond this point, the correlation starts to rise slightly but remains negative, indicating a persistent inverse relationship between past sentiment and present CPI. The computed p-values for these correlations were all below 0.01, confirming statistical significance at conventional confidence levels, such as 95% or 99%. This suggests that the observed relationship is unlikely to be due to random variation. The negative correlation implies that higher sentiment values in the past tend to be associated with lower CPI values in the future. This may indicate that optimistic sentiment precedes periods of lower inflation, whereas pessimistic sentiment is followed by higher inflationary pressures. The strongest relationship, observed at a 400-day lag, suggests a possible time frame for sentiment shifts to influence price levels. In short, the past sentiment index exhibits a significant and inverse relationship with future CPI, with the most pronounced effect occurring around a 400-day lag.

In summary, our findings confirm that the methodology used to compute the sentiment index is empirically sound, and the stable negative correlation between the sentiment index and Total CPI indicates that the sentiment index could be a useful, predictor of CPI movements. This is validated further by comparing loss landscape with or without sentiment index during training in [sec:loss_landscape]Sect 5.3.2.

### 3.3. Data preprocessing and augmentation

Both CPI and news sentiment index are normalized to ensure consistent scaling. For this, all values were normalized to a 0–1 range using: $x=\frac{x-x_{\min}}{x_{\max}-x_{\min}}$. Sentiment scores underwent a 7-day smoothing process. Training and test datasets were processed separately to maintain temporal integrity and prevent data leakage.

**Fig 5:**
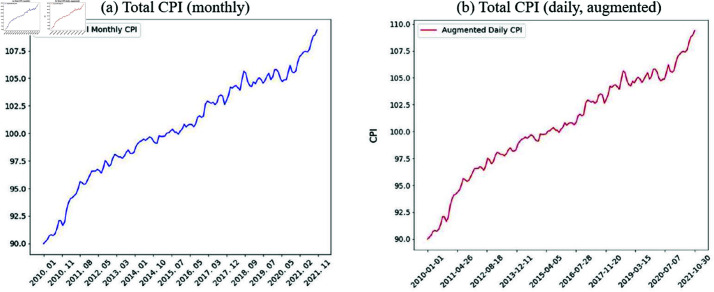
(a) Original CPI data at monthly frequency; (b) Daily frequency data generated via linear interpolation. Linear interpolation augments data, making it suitable for deep learning models while preserving trends and patterns.

We augmented the CPI indices through linear interpolation, converting monthly observations into daily values. Linear interpolation can preserve the shape of the original data without bias while generating daily data from the monthly data [53, 54]. Table 3 shows the detailed statistics for original and augmented data. Fig 5(a) presents the original CPI data, while Fig 5(b) shows the daily data augmented from it using linear interpolation. The comparison reveals that the linearly interpolated data maintained the same trend as the original.

**Table 3:** Summary statistics for augmented vs. original.

Dataset	Split	Count	Mean	St. Dev.	Med.	Min.	Max.	Start Date	End Date
Augmented	Train	3042	98.29	3.65	99.11	89.97	104.34	2010-01-01	2018-04-30
	Val.	365	104.67	0.46	104.59	103.93	105.65	2018-05-01	2019-04-30
	Test	915	106.15	1.34	105.56	104.56	109.48	2019-05-01	2021-10-31
Original	Train	100	98.22	3.68	99.09	89.97	104.29	2010-01-01	2018-04-01
	Val.	12	104.64	0.49	104.59	103.93	105.65	2018-05-01	2019-04-01
	Test	30	106.08	1.29	105.55	104.56	108.95	2019-05-01	2021-10-01

In linear interpolation, if the function values at points *p*_1_ and *p*_2_ are *f*(*p*_1_) and *f*(*p*_2_), respectively, then the estimated value f(p) at any point *p* between *p*_1_ and *p*_2_ is given by:

$$f(p)=\frac{d_{2}}{d_{1}+d_{2}}f(p_{1})+\frac{d_{1}}{d_{1}+d_{2}}f(p_{2})$$ (6)

where *d*_1_ is the distance between *p* and *p*_1_, and *d*_2_ is the distance between *p* and *p*_2_.

As a result of this augmentation, the original 143 months of data were expanded into 4,323 daily data points. Table 3 presents the summary statistics for the augmented and original datasets. To maintain brevity, only the Total CPI values are presented.

The predicted daily values for each month were averaged and compared with the corresponding true reported monthly values.

### 3.4. Creating sliding window dataset

For data preparation, we constructed a sliding window dataset based on the methodology of Chou *et al*. [14]. To ensure consistency, we adopted the same lookback window of 10 months used in their study.

**Fig 6:**
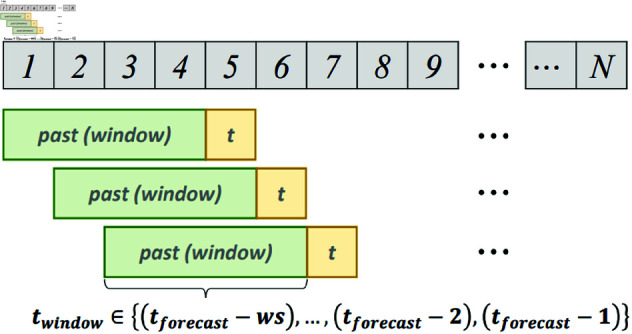
Sliding window dataset construction process.

As illustrated in Fig 6, a fixed-size window moves stepwise across the time series, capturing past observations (green) as input and associating them with the target value (yellow). The input sequence spans $\left(t_{\text{forecast}}-ws\right)$ to $\left(t_{\text{forecast}}-1\right)$, where *ws* is the window size. This approach preserves temporal dependencies, facilitating effective forecasting.

Once constructed, the dataset is partitioned into training, validation, and evaluation sets. The entire dataset spans 143 months, from January 1, 2010, to November 1, 2021. The training period, which accounts for 75% of the data, covers January 1, 2010, to April 30, 2018. The validation period comprises 10% of the data and spans May 1, 2018, to April 30, 2019. The test period, covering the remaining 15% of the data, extends from May 1, 2019, to October 31, 2021. This temporal separation ensures that the model is trained on historical data, validated on an intermediate period, and tested on the most recent data for robust evaluation.

### 3.5. Proposed CNN-LSTM model for forecasting

**Fig 7:**
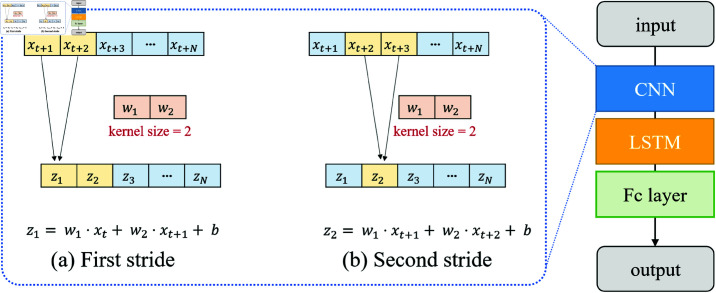
Proposed CNN-LSTM model for time-series forecasting. The left panel illustrates feature extraction via 1D CNN strides with a kernel size 2. The right panel shows the model architecture, combining CNN for local patterns, LSTM for long-term dependencies, and fully connected layers for prediction.

Since the CPI-related numerical features and sentiment score are one-dimensional (1D), feature extraction was performed using a 1D CNN layer. The hyperparameters were configured with a kernel size of 2 and a stride of 1, following [30]. For a kernel size of 2, the 1D CNN layer includes two weight parameters (*w*_1_, *w*_2_) and one bias parameter (*b*). Fig 7 demonstrates the kernel shifting process, where the kernel moves from left to right with a step size of 1 at each iteration, illustrating how the features at input values *x*_*t*_ and *x*_*t* + 1_ are extracted from the data. The formulas for computing intermediate features *z*_1_ and *z*_2_ are given as follows:

$$z_{1}=w_{1}\cdot x_{t}+w_{2}\cdot x_{t+1}+b$$ (7)

$$z_{2}=w_{1}\cdot x_{t+1}+w_{2}\cdot x_{t+2}+b$$ (8)

Here, *d*_1_ is the feature extracted from *x*_*t*_ and *x*_*t* + 1_, and *d*_2_ is the feature extracted from *x*_*t* + 1_ and *x*_*t* + 2_. The weights *w*_1_ and *w*_2_ are applied to the inputs within the kernel, and *b* is the bias term added to the weighted sum. [sec:cnninternal]Sect 5.3.1 discusses the effect of this layer on features.

After the features are extracted by the 1D CNN layer, the LSTM layer processes the information sequentially. First, the forget gate layer evaluates whether certain information should be discarded. Next, unnecessary information is removed through the input gate layer, and the cell state is updated accordingly. Finally, the output gate layer generates the actual forecasting result based on the updated cell state information.

$$\text{Forget gate:}{𝐟}_{t}=\sigma ({𝐖}_{f}{𝐱}_{t}+{𝐔}_{f}{𝐡}_{t-1}+{𝐛}_{f})$$ (9)

$$\text{Input gate:}{𝐢}_{t}=\sigma ({𝐖}_{i}{𝐱}_{t}+{𝐔}_{i}{𝐡}_{t-1}+{𝐛}_{i})$$ (10)

$$\text{Input modulation gate:}{𝐠}_{t}=\tanh({𝐖}_{g}{𝐱}_{t}+{𝐔}_{g}{𝐡}_{t-1}+{𝐛}_{g})$$ (11)

$$\text{Updated cell state:}{𝐜}_{t}={𝐟}_{t}⊙{𝐜}_{t-1}+{𝐢}_{t}⊙{𝐠}_{t}$$ (12)

$$\text{Output gate:}{𝐨}_{t}=\sigma ({𝐖}_{o}{𝐱}_{t}+{𝐔}_{o}{𝐡}_{t-1}+{𝐛}_{o})$$ (13)

$$\text{Output of the memory cell:}{𝐡}_{t}={𝐨}_{t}⊙\tanh({𝐜}_{t}),$$ (14)

where σ(·) denotes the element-wise sigmoid function tanh(·) denotes the element-wise hyperbolic tangent function ⊙ denotes the Hadamard (element-wise) product ${𝐖}_{i},{𝐖}_{g},{𝐖}_{f},{𝐖}_{o}$ are input weight matrices, ${𝐔}_{i},{𝐔}_{g},{𝐔}_{f},{𝐔}_{o}$ are recurrent weight matrices, and ${𝐛}_{i},{𝐛}_{g},{𝐛}_{f},{𝐛}_{o}$ are bias vectors.

## 4. Experimental setup

### 4.1. Input and forecasting horizon settings

For time series forecasting, determining appropriate temporal window settings is crucial for model performance. Our model used a lookback window of 10 months, processing 310 days (31 days × 10 months) of historical data for each prediction. This window length was determined through empirical experiments that evaluated how different historical spans affected prediction accuracy. A shorter window failed to capture sufficient historical patterns leading to information loss, while extending the window beyond 10 months added computational complexity without yielding meaningful improvements in forecast accuracy.

The model generated predictions for a 1-month horizon (31 days), with daily forecasts averaged to produce monthly values that aligned with the official CPI reporting frequency. This averaging approach addressed the mismatch between our model’s daily granularity and the monthly cadence of CPI publications, ensuring meaningful performance evaluation while maintaining the model’s ability to capture daily market dynamics.

### 4.2. Hyperparameter settings

We conducted systematic hyperparameter tuning using a combination of Optuna-based Bayesian optimization [55] and grid search. While Optuna enables faster and automated optimization, it inherently reduces interpretability and direct control over the search process. To address this, we adopted a hybrid strategy: Optuna was used for all parameters except hidden unit size, which was tuned via grid search to maintain interpretability.

Given the stochastic nature of training, each configuration was evaluated using 20 random seeds. To mitigate variance, we based our decisions on the five middle-ranked results. Dropout rates were optimized within a range of 0.1 to 0.7, with Optuna converging to 0.6 as the optimal setting, balancing regularization and performance. The learning rate was also optimized via Optuna to ensure a trade-off between fast convergence and stability.

For CNN hyperparameters, we followed [30], setting the kernel size to 2 and stride to 1. Hidden unit size was tuned separately using a grid search over 64, 128, 256, and 512 units, revealing that 512 units provided the best performance. Models with fewer units struggled to capture long-term dependencies, while larger architectures beyond 512 units increased computational costs without meaningful improvements.

Training optimization was performed using the Adam optimizer [56], selected for its adaptive learning rates and stability. To prevent overfitting, we implemented early stopping with patience of 1, halting training when validation performance plateaued. This conservative approach avoided unnecessary computations while allowing effective learning. All baseline models were configured using these optimized hyperparameters to ensure fair comparison.

### 4.3. Evaluation metrics

We evaluate the model’s performance using several metrics: RMSE, Mean Absolute Error (MAE), Mean Absolute Percentage Error (MAPE), and Symmetric Mean Absolute Percentage Error (SMAPE). In the following equations, *n* represents the total number of test data points, *y*_*i*_ represents the actual values of the test data, and ${\hat{y}}_{i}$ represents the predicted values of the test data. The RMSE calculates the square root of the mean of the squared residuals. The formula is as follows:

$$\text{RMSE}=\sqrt{\frac{1}{n}\sum_{i=1}^{n}{\left(y_{i}-{\hat{y}}_{i}\right)}^{2}}$$ (15)

The MAE can be more robust as it is less sensitive to outliers than the RMSE. The formula is as follows:

$$MAE=\frac{1}{n}\sum_{i=1}^{n}\left|y_{i}-{\hat{y}}_{i}\right|$$ (16)

The MAPE is calculated by dividing the difference between the actual and predicted values by the actual value and calculating the relative proportion of the error to the true value between 0 and 100%. Subsequently, the absolute values of the previously calculated values are averaged. The formula is as follows:

$$MAPE=\frac{1}{n}\sum_{i=1}^{n}\left|\frac{y_{i}-{\hat{y}}_{i}}{y_{i}}\right|\times 100$$ (17)

The SMAPE was developed to address that MAPE cannot be calculated when the actual value is 0. In addition, even if the absolute value of MAPE has the same error, SMAPE limits overestimation by considering the relationship between the actual and predicted values. The formula is as follows:

$$SMAPE=\frac{1}{n}\sum_{i=1}^{n}\frac{\left|y_{i}-{\hat{y}}_{i}\right|}{\left(|y_{i}|+|{\hat{y}}_{i}|\right)/2}\times 100$$ (18)

The Normalized Root Mean Square Error (NRMSE) provides a scale-independent measure of error by normalizing RMSE with respect to the standard deviation of the evaluation dataset. This normalization enables direct comparison across datasets with different scales. The formula for NRMSE is:

$$NRMSE=\frac{RMSE}{stdev(y)}$$ (19)

where stdev(y) is the standard deviation of the actual values in the evaluation dataset. By scaling RMSE with the standard deviation of the target variable (Total CPI), NRMSE provides a standardized evaluation of forecasting error. This adjustment contextualizes model performance relative to the natural variability of CPI, offering a clearer perspective on the practical significance of the error.

In this study, the standard deviation of the CPI evaluation set (test set excluding the lookback period) was 1.212, as shown in Table 4, representing the inherent fluctuations in the data. Models with NRMSE values near or below this threshold effectively capture real-world CPI variations, demonstrating their practical forecasting utility.

**Table 4:** Summary of evaluation range statistics.

Statistic	Value
Mean	105.981
Median	105.512
Standard Deviation	1.212

### 4.4. Computational setup

The experimental framework was developed in Python 3.9, leveraging the PyTorch library for deep learning implementations. The computational infrastructure consisted of an NVIDIA L40S GPU coupled with an AMD EPYC 9654 96-core processor and 1 TiB RAM. To maintain the highest level of numerical precision during model comparisons, all computations were performed using double-precision floating-point (float64) operations.

## 5. Experimental results

### 5.1. Performance comparison with baseline methods

#### 5.1.1. Description of baseline models.

To assess the effectiveness of our proposed models, we compare their performance against standard machine learning and deep learning baselines. The evaluation includes both variations of our model and established forecasting approaches.

Three ablated versions of our model serve as internal baselines to analyze the contribution of different components. The *wo. CPI-multi* variant excludes all auxiliary CPI-related variables, using only the Total CPI as input. The *wo. senti.* variant removes sentiment information to evaluate its impact on prediction accuracy. The *wo. aug.* variant omits data augmentation while retaining both CPI-related variables and sentiment scores, allowing us to isolate the effect of augmentation on model performance.

For external baselines, we incorporate four machine learning models that are commonly applied to multivariate time-series forecasting. Lasso regression [43] is included for its feature selection capability via *L1* regularization, which helps identify the most influential CPI components. However, its assumption of linear relationships limits its ability to capture complex dependencies. Support Vector Regression (SVR) [57] is selected due to its ability to model nonlinear relationships through kernel functions, though its computational inefficiency and lack of sequential modeling make it less suitable for large-scale time-series forecasting. Random Forest [58] is chosen for its robustness against overfitting and its ability to capture multivariate interactions. While Random Forest effectively handles structured features, its reliance on independently grown decision trees prevents it from modeling temporal dependencies. XGBoost [59], an optimized gradient boosting algorithm, is included for its strong performance in structured regression tasks, leveraging iterative residual minimization to improve accuracy. However, XGBoost does not inherently model sequential dependencies, making it less flexible for raw time-series data.

Deep learning baselines are selected to evaluate the impact of different architectures on CPI forecasting and to analyze the impact of their components. A Feedforward Neural Network (FFNN) provides a baseline for nonlinear modeling but is unable to retain sequential information due to the absence of memory mechanisms. Gated Recurrent Units (GRU) [60] offer a more computationally efficient alternative to LSTMs [25], enabling a comparison between efficiency and long-term dependency modeling. LSTM networks are included as a widely used benchmark in time-series forecasting, given their ability to retain temporal dependencies over extended periods. Additionally, we evaluate an AttentionLSTM [61], which replaces CNN-based local feature extraction with an attention mechanism. This allows us to assess whether direct multivariate attention is a viable alternative to convolutional feature extraction for capturing dependencies in CPI data.

#### 5.1.2. Performance breakdown.

Table 5 compares our model against various baseline methods, including Lasso, Random Forest, XGBoost, SVR, and deep learning models such as Feedforward Neural Networks (FFNN), GRU, LSTM, and AttentionLSTM, as outlined in [sec:descriptionmodels]Sect 5.1.1.

**Table 5:** Performance comparison of baseline models and ablation variants, showing the impact of excluding key elements (*CPI-multi*, *sentiment index*, and *data augmentation*).

	RMSE	NRMSE	MAE	MAPE	SMAPE
Lasso (*wo. CPI-multi.*)	7.3934	6.1002	7.3605	6.9046	7.1534
Lasso (*wo. senti.*)	6.8901	5.6848	6.8513	6.4261	6.6415
Lasso (*wo. aug.*)	6.8954	5.6892	6.8570	6.4315	6.6472
Lasso (full)	7.3934	6.1002	7.3605	6.9046	7.1534
Random Forest (*wo. CPI-multi.*)	3.9185	3.2330	3.8772	3.6354	3.7040
Random Forest (*wo. senti.*)	3.9128	3.2283	3.8704	3.6290	3.6974
Random Forest (*wo. aug.*)	3.9362	3.2476	3.8887	3.6460	3.7152
Random Forest (full)	3.9071	3.2236	3.8659	3.6249	3.6931
SVR (*wo. CPI-multi.*)	6.2694	5.1728	6.2380	5.8514	6.0293
SVR (*wo. senti.*)	6.2137	5.1268	6.1801	5.7970	5.9716
SVR (*wo. aug.*)	6.4050	5.2847	6.3729	5.9780	6.1637
SVR (full)	6.2694	5.1728	6.2380	5.8514	6.0293
XGBoost (*wo. CPI-multi.*)	3.9783	3.2824	3.9107	3.6663	3.7370
XGBoost (*wo. senti.*)	3.8363	3.1652	3.7922	3.5563	3.6220
XGBoost (*wo. aug.*)	3.6482	3.0100	3.5801	3.3555	3.4148
XGBoost (full)	3.9783	3.2824	3.9107	3.6663	3.7370
FFNN (*wo. CPI-multi.*)	0.3298	0.2722	0.2792	0.2613	0.2618
FFNN (*wo. senti.*)	0.4357	0.3595	0.3779	0.3537	0.3546
FFNN (*wo. aug.*)	6.0736	5.0112	6.0033	5.6283	5.8127
FFNN (full)	0.4434	0.3659	0.3869	0.3622	0.3632
GRU (*wo. CPI-multi.*)	1.3995	1.1547	1.3685	1.2822	1.2908
GRU (*wo. senti.*)	0.3468	0.2862	0.3042	0.2865	0.2860
GRU (*wo. aug.*)	14.2467	11.7546	14.1988	13.3210	14.2830
GRU (full)	0.8406	0.6935	0.7657	0.7156	0.7189
LSTM (*wo. CPI-multi.*)	1.6410	1.3539	1.5773	1.4766	1.4885
LSTM (*wo. senti.*)	0.5244	0.4327	0.4822	0.4536	0.4521
LSTM (*wo. aug.*)	15.6921	12.9472	15.6459	14.6795	15.8490
LSTM (full)	0.5544	0.4574	0.4728	0.4416	0.4431
AttentionLSTM (*wo. CPI-multi.*)	1.5721	1.2971	1.3360	1.2460	1.2567
AttentionLSTM (*wo. senti.*)	0.7133	0.5886	0.5768	0.5436	0.5425
AttentionLSTM (*wo. aug.*)	14.4642	11.9341	14.4149	13.5236	14.5111
AttentionLSTM (full)	0.9357	0.7721	0.8083	0.7549	0.7590
**Ours** (*wo. CPI-multi.*)	0.2074	0.1711	0.1917	0.1795	0.1796
**Ours** (*wo. senti.*)	0.2065	0.1704	0.1850	0.1743	0.1741
**Ours** (*wo. aug.*)	1.4570	1.2022	1.2375	1.1540	1.1634
**Ours** (full)	0.2012	0.1660	0.1460	0.1373	0.1372

**Fig 8:**
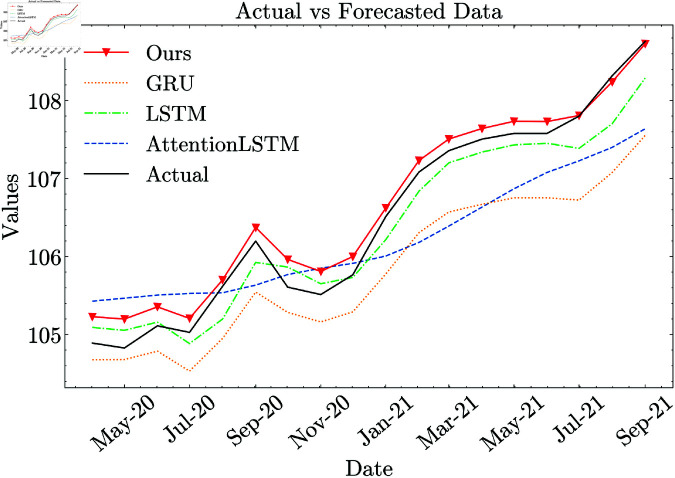
Comparison of model performance against baseline deep learning methods.

The performance of Ours (full) demonstrates substantial improvements over both machine learning (ML) and deep learning (DL) models in terms of RMSE, NRMSE, MAE, MAPE, and SMAPE. Specifically, RMSE is reduced by 96.27% compared to ML models and by 66.19% compared to DL models. Relative to ML models, Ours (full) achieves a 97.27% decrease in MAE, 97.26% in MAPE, and 97.34% in SMAPE. When compared to DL models, it still maintains a strong advantage, reducing MAE by 71.70%, MAPE by 71.53%, and SMAPE by 71.67%. Fig 8 shows that our proposed CNN-LSTM model with full configuration’s forecast values are closer to the actual values than those of other models.

In absolute terms, Ours (full) achieves a reduction of 5.198 in MAE, 4.875 in MAPE, and 5.016 in SMAPE relative to ML models. When compared to DL models, the reductions remain significant but smaller, with decreases of 0.370 in MAE, 0.345 in MAPE, and 0.347 in SMAPE. Additionally, Ours (full) reduces NRMSE by 4.279 compared to ML models, representing a substantial improvement. When compared to DL models, the improvement remains significant, with a reduction of 0.325.

These results highlight the superiority of Ours (full), particularly against ML models, where the reduction exceeds 96% on average. Even in comparison to DL models, the model achieves a notable 66% improvement in RMSE.

As shown in Table 6, the Wilcoxon Signed-Rank test [62] results indicate that the null hypothesis, which assumes no difference in RMSE performance between our model and the compared models, is rejected at a significance level below 0.05.

**Table 6:** Wilcoxon Signed Rank test results.

Model	*p*-value
AttentionLSTM	0.037
GRU	0.037
LSTM	0.037
FFNN	0.037

### 5.2. Discussion

#### 5.2.1. Ablation study: Contribution of key components.

**Fig 9:**
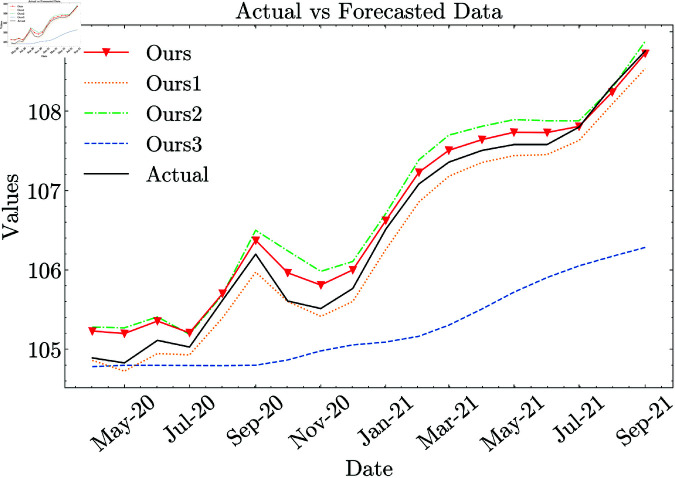
Performance comparison of the proposed model with ablation variants, highlighting the impact of individual components. Ours1, Ours2, and Ours3 represent the proposed model *without CPI-multi*, *without the sentiment index*, and *without data augmentation*, respectively.

The impact of individual components (*CPI-multi*, *sentiment index*, and *data augmentation*) is analyzed through ablation studies, as presented in Table 5. Fig 9 shows that the fully configured CNN-LSTM model we propose produces forecast values that align more closely with the actual values compared to ablated models.

The effect of different components on RMSE varies between machine learning (ML) and deep learning (DL) models. In ML models, such as Lasso, Random Forest, SVR, and XGBoost, removing the *CPI-multi* variable does not significantly alter RMSE, which remains nearly unchanged. Surprisingly, excluding sentiment data results in a modest improvement, slightly reducing RMSE. Similarly, removing augmentation leads to a slight RMSE reduction of about 3.1%. These observations indicate that ML models are largely insensitive to *CPI-multi* but show minor improvements when sentiment and augmentation are removed.

Conversely, DL models, including FFNN, GRU, LSTM, AttentionLSTM, and the proposed model, exhibit a stronger dependency on certain components. Removing *CPI-multi* results in a substantial RMSE increase of 73%, indicating its importance. nevertheless, eliminating sentiment data slightly improves RMSE on average, except for our proposed CNN-LSTM model and Random Forest suggesting that for some models, sentiment may introduce noise. The most pronounced effect is observed with augmentation removal, which leads to an average RMSE increase by a factor of 17.45, underscoring its crucial role in ensuring robust performance in deep learning models.

These findings suggest that ML models are largely unaffected by *CPI-multi* and experience slight benefits from removing sentiment and augmentation. In contrast, DL models depend heavily on augmentation and CPI-multi, as their removal substantially degrades performance.

The improved performance of our model due to sentiment unlike other DL models may be attributed to the use of a 1D CNN in our proposed CNN-LSTM architecture, which likely helps smooth noisy sentiment data, as shown in [sec:cnninternal]Sect 5.3.1. By reducing noise in the input features, the model can extract more relevant patterns, thereby outperforming models that process raw sentiment data directly. This may also explain why removing sentiment data has minimal to adverse impact on baseline models but affects our model, as noise mitigation enhances its effectiveness.

#### 5.2.2. NRMSE: Evaluating RMSE in absolute terms and forecast reliability.

As defined in Eq. 19, NRMSE is the normalized RMSE, obtained by dividing RMSE by the standard deviation of the evaluation set. When NRMSE is below 1, it indicates that the forecasting error is smaller than the variability in the target values, ensuring that the forecast retains practical relevance.

This aligns with established methodologies, such as those presented by Chai *et al*. [63], which emphasize the importance of normalizing error metrics to account for differences in data scale and variance. By incorporating NRMSE, our evaluation provides a standardized measure of forecasting accuracy, allowing direct comparisons between model predictions and the natural fluctuations in CPI.

The effectiveness of our approach is reflected in the NRMSE results. Our CNN-LSTM model achieved an NRMSE of 0.1660, approximately one-sixth of the evaluation set’s standard deviation, highlighting its ability to generate highly accurate forecasts relative to CPI’s inherent variability. In contrast, most machine learning models, including Random Forest, XGBoost, and Lasso, recorded NRMSE values above 1, underscoring their limitations in capturing CPI trends with the same level of precision.

Ultimately, the low NRMSE of our model highlights its practical utility in generating precise and reliable CPI forecasts, addressing both statistical and real-world forecasting needs.

### 5.3. Supplementary studies

#### 5.3.1. Evaluating the noise reduction capability of 1D CNN layers: Internal analysis.

**Fig 10:**
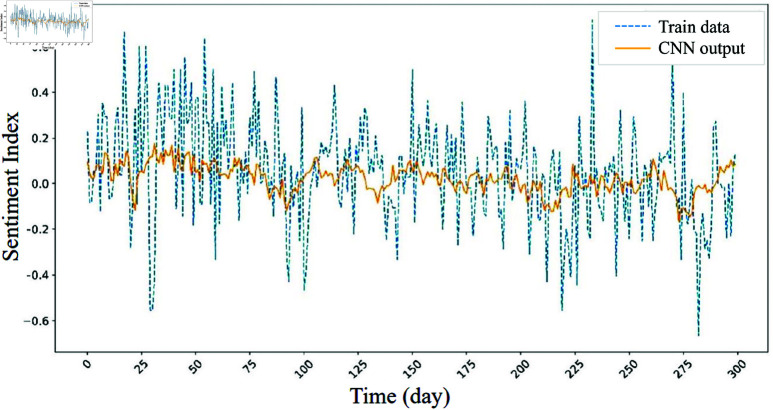
Internal analysis of the 1D CNN layer: comparison of input features before and after processing through the layer.

The intermediate output visualization in Fig 10 offers insights into the CNN-LSTM model’s internal processing and validates the effectiveness of the hybrid approach. To evaluate the contribution of the CNN and LSTM layers, we applied the method of Kim *et al*. [64]. The results show that the CNN layer effectively reduces noise in the time series data while preserving essential trends.

Fig 10 demonstrates that the sentiment index data, after passing through the CNN layer, retains its overall temporal pattern with reduced noise. Since sentiment index data contain irregular temporal variations, maintaining these characteristics is crucial for accurate prediction. The processed data then serve as input to the LSTM layer, facilitating more effective training.

#### 5.3.2. Utility of the sentiment index: Analyzing the loss landscape of the CNN-LSTM hybrid model.

The loss landscape analysis provides strong evidence that incorporating the sentiment index enhances the CNN-LSTM hybrid model. This addition not only improves optimization stability but also strengthens model performance in sentiment analysis tasks.

To evaluate its impact, we visualized the loss landscape using the filter normalization method proposed by Li *et al*. [51]. This technique facilitates meaningful comparisons by normalizing convolutional filters and fully connected neurons separately, ensuring a fair representation of the loss curvature.

**Fig 11:**
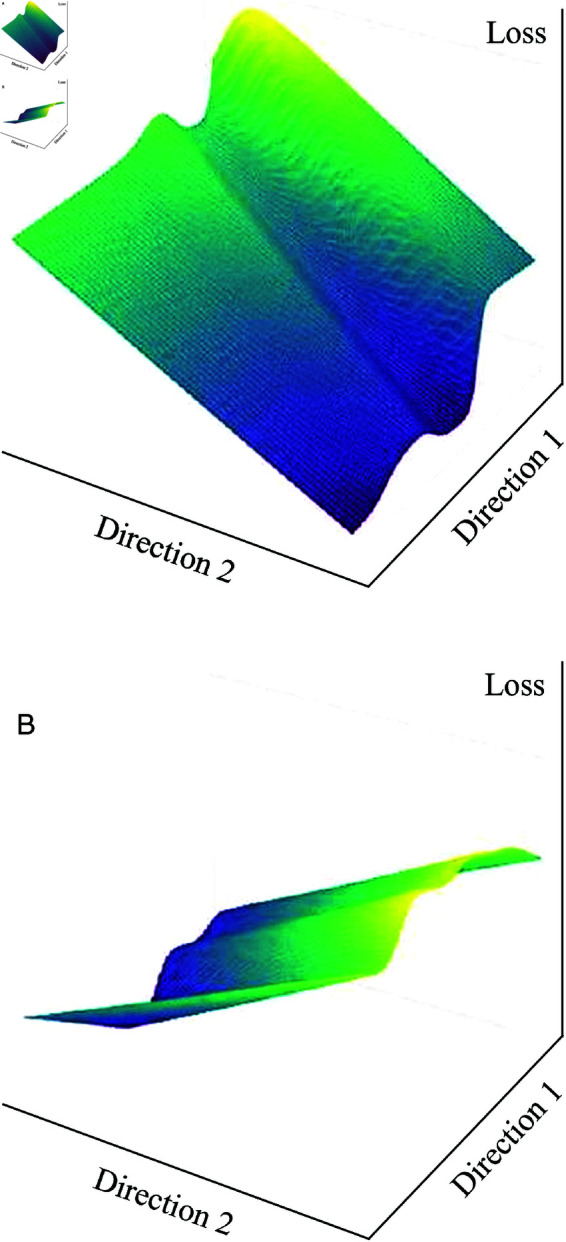
Loss surface without sentiment index. (a) Loss surface without sentiment index. (b) Loss surface with sentiment index.

We compared two scenarios: the CNN-LSTM model without the sentiment index (Fig 11a) and with it (Fig 11b). The results highlight key differences. First, the loss landscape in Fig 11b is noticeably smoother, indicating more stable optimization. Second, this smoothness suggests that gradient descent converges more efficiently, making training more robust. Third, a less chaotic loss landscape reduces the risk of getting stuck in suboptimal local minima while increasing the likelihood of reaching a well-generalized solution.

Li *et al*. [37] argue that smoother loss landscapes lead to better generalization and reduced overfitting. Our findings align with this perspective, demonstrating that the sentiment index improves optimization dynamics, enhances predictive performance, and facilitates a more reliable training process.

## 6. Future implications and work

To the best of our knowledge, there has been limited exploration of time series augmentation using simple, non-parametric methods such as linear interpolation. Our findings demonstrate that this straightforward approach can significantly enhance forecasting performance, highlighting the potential of lightweight augmentation strategies in time series analysis.

While our model demonstrates strong predictive performance, several aspects require deeper investigation. For instance, given the substantial performance gains observed, further research into augmentation methods could be valuable in advancing forecasting techniques. For instance, integrating more sophisticated augmentation strategies that incorporate domain-specific priors may yield further improvements. Additionally, the inclusion of complementary data sources, such as macroeconomic indicators or domain knowledge, could further enhance model robustness and better align data-driven forecasting with real-world decision-making.

In addition, a more rigorous investigation into the variability of component contributions could further clarify their impact across different models and datasets. For instance, our future work should explore the stability of these effects under a broader range of hyperparameter configurations and across multiple datasets. More systematic feature attribution techniques, such as SHAP values, or permutation importance could further refine our understanding of each component’s role in predictive performance.

By addressing these considerations, future research will provide a more comprehensive understanding of the role of augmentation and other model components in forecasting, paving the way for more effective and interpretable predictive models.

## 7. Conclusion

We proposed a new CPI forecasting framework using a hybrid CNN-LSTM model with augmented multivariate data and a sentiment score for accurate CPI prediction. Compared to previous studies, the proposed model demonstrated a notable performance improvement. The hybrid CNN-LSTM model effectively addresses challenges in CPI prediction through four key techniques. First, multivariate time series prediction incorporates a variety of complex variables into the deep learning model. Second, data augmentation via linear interpolation mitigates the event of limited data availability, while preserving the original data trend without bias. Third, sentiment analysis of news articles captures unstructured data from global events like COVID-19, politics, and natural disasters, transforming them into a sentiment score. Finally, the CNN-LSTM hybrid approach extracts features from complex CPI and sentiment index through the CNN layer, which reduces noise and retains temporal, local, and global data characteristics, followed by accurate CPI prediction using the LSTM layer. By incorporating external events through sentiment analysis, this model provides a flexible prediction framework responsive to unexpected occurrences such as COVID-19.
